# Clinical applicability of short form of Bruininks-Oseretsky Test of Motor Proficiency Second Edition in patients after treatment of acute lymphoblastic leukemia

**DOI:** 10.3389/fped.2023.1071572

**Published:** 2023-04-03

**Authors:** Tereza Šnajdrová, Eliška Patrmanová, Filip Jevič, Karolína Bořilová, Monika Hrdoušková, Martin Musálek

**Affiliations:** ^1^ Department of Rehabilitation and Sports Medicine, Second Faculty of Medicine, Charles University and Motol University Hospital, Prague, Czechia; ^2^ Faculty of Physical Education and Sport, Charles University, Prague, Czechia

**Keywords:** acute lymphoblastic leukemia, Bruininks-Oseretsky Test of Motor Proficiency Second Edition, motor development, motor skills, motor assessment, standard score

## Abstract

**Introduction:**

Acute lymphoblastic leukaemia (ALL) ranks among paediatrics' most common oncological malignancies. Monitoring motor performance levels associated with self-sufficiency in the everyday activities of ALL patients is extremely important during treatment. The motor development of children and adolescents with ALL is most often assessed using the Bruininks-Oseretsky Test of Motor Proficiency Second Edition (BOT-2) complete form (CF) with 53 items or the short form (SF) with 14 items. However, there is no evidence in research that BOT-2 CF and SF give comparable results in the population of patients with ALL.

**Objective:**

This study aimed to determine the compatibility of motor proficiency levels achieved from BOT-2 SF and BOT-2 CF in ALL survivors.

**Materials and Method:**

The research sample consists of *n* = 37 participants (18 girls, 19 boys) aged 4–21 years (10.26, ± SD 3.9) after treatment for ALL. All participants passed BOT-2 CF and were at least 6 months and a maximum of 6 years from the last dose of vincristine (VCR). We used ANOVA with repeated measures, considering the sex, intra-class correlation (ICC) for uniformity between BOT-2 SF and BOT-2 CF scores and Receiving Operating Characteristic.

**Results:**

BOT-2 SF and BOT-2 CF assess the same underlying construct, and BOT-2 SF and CF standard scores have good uniformity: ICC = 0.78 for boys and ICC = 0.76 for girls. However, results from ANOVA showed that the participants achieved a significantly lower standard score in SF (45.1 ± 7.9) compared to CF (49.1 ± 9.4) (*p* < 0.001; Hays *ω*^2^ = 0.41). ALL patients performed the worst in Strength and Agility. According to the ROC analysis, BOT-2 SF obtains acceptable sensitivity (72.3%) and high specificity (91.9%) with high accuracy of 86.1%, and the fair value of the Area Under the Curve (AUC) = 0.734 CI95% (0.47–0.88) in comparison to BOT-2 CF.

**Conclusions:**

To reduce the burden on ALL patients and their families, we recommend using BOT-2 SF instead of BOT-2 CF as a useful screening tool. BOT-SF can replicate motor proficiency with as high probability as BOT-2 CF but systematically underestimates motor proficiency.

## 1. Introduction

Acute lymphoblastic leukaemia (ALL) is one of the most common pediatric malignancies (1–3), and the number of patients has been increasing over recent decades (2, 4). The incidence is 3–4 new cases/100,000 children in one year, peaking in the second and fifth years of life (5). This disease is typified by the uncontrolled proliferation of lymphocyte precursors and occurs in the bone marrow. Due to the ideal vascular supply of the marrow, it quickly spreads to other tissues and organs (6, 7). Treatment of children with ALL is complex (3) and has several side effects (2, 8). It has developed over the last fifty years and is very successful, with the five-year survival rate having increased from 3% in 1964 to 90% today (8, 9). The standardized treatment protocol includes systematic chemotherapy, usually with cytostatic corticosteroids (3). In addition to chemotherapy, radiotherapy and bone marrow transplantation may be added (10, 11).

The patients undergo long periods of examinations and hospitalizations (1–3, 12–15). During and after the treatment, children with ALL are at risk for skeletal (6, 7, 16, 17), neuromuscular (2, 6, 7), and cardiopulmonary impairments that interfere with physical function (18–20). Therefore, increased attention is directed to adequate rehabilitation and assessment methods that meet the individual needs of these children (18). For objective evaluation, paediatricians, physiotherapists, and physical education teachers frequently use the Bruininks-Oseretsky Test of Motor Proficiency Second edition (BOT-2) (17, 21–25). BOT-2 measures gross and fine motor proficiency in typically developing children and in children with developmental disabilities. The children are aged between 4 and 21 (26, 27). The test is available in two forms, complete form (CF) and short form (SF) (28).

BOT-2 is a reliable and valid assessment tool suitable for many research designs (29, 30). BOT-2 CF requires setup and 40–60 min to administer per child (29). This assessment is an additional burden for a child and even their parents. Therefore, many experts call for greater use of BOT-2 SF, whose advantage is a shorter administration time of 15 to 20 min (28, 31). BOT-2 CF and BOT-2 SF have high reliability (0.9–0.97), and there is a strong correlation between these two test forms (*r* = 0.80–0.87) (29–32). However, the strong correlation between BOT-2 CF and BOT-2 SF does not mean that both versions give the same estimation of motor proficiency level (26, 27, 33).

Although Radanović et al. (28) conclude that BOT-2 SF is an adequate tool for assessing motor proficiency in healthy children, it is still unclear whether BOT-2 SF and BOT-2 CF provide comparable information about the level of motor proficiency (31). Jirovec et al. (31) found that BOT-2 SF systematically underestimates the degree of motor proficiency compared to BOT-2 CF and that both forms shared just 57% of the variance. In addition, other studies point to BOT-2 SF's psychometric shortcomings in the majority of tasks related to factorial validity or low correlations between subtests and final scores (34). On the other hand, skilled clinicians claim BOT-2 SF is useful for identifying motor skills deficits in individuals with mild to moderate motor control problems (27, 28, 31).

Many studies use BOT-SF in children with various diagnoses but with different outcomes from the diagnoses and reached by the studies. Mancini et al. (35) assess children with attention deficit and hyperactivity disorder (ADHD). The results indicate that BOT-2 SF overestimates the child's motor proficiency compared to that shown by BOT-2 CF. Therefore, BOT-2 SF is not recommended as a screening or diagnostic tool in children with ADHD. Cairney et al. (36) reveal that BOT-2 SF seems to be a reasonable alternative to Movement Assessment Battery for Children Second Edition (MABC-2) for clinical assessment of children with developmental coordination disorder (DCD). Furthermore, in a pilot study on 10 youths with Down syndrome, Nocera et al. (37) found excellent reliability (ICC = 0.86, 95% CI: 0.54 to 0.96, *p* < 0.001, SEM = 1.46; percentile rankings: ICC = 0.84, 95% CI 0.50 to 0.96, *p* = 0.001, SEM = 0.64). On the other hand, Yeh et al. (38), who investigate preschool children with strabismus, suggest assessing the motor competency to achieve more accurate information using BOT-2 CF rather than BOT-2 SF.

The usefulness of the BOT-2 results remains an open issue. Moreover, no comparative studies investigate the compatibility of results between BOT-2 CF and BOT-2 SF and the sensitivity and specificity of BOT-2 SF in patients with ALL. This study aims to verify whether BOT-2 SF gives comparable results to BOT-2 CF in ALL patients and has sufficient sensitivity and specificity in motor proficiency categorization. Using BOT-2 SF as a sufficiently sensitive and reliable tool would enable faster and more user-friendly method of diagnosis changes in motor proficiency during the recovery of ALL patients.

## 2. Materials and methods

The procedures involved in our study follow the ethical standards of the Czech National Committee on Human Experimentation and the Helsinki Declaration of 1975, as revised in 2000. The Ethics Committee on the Research Project of Motol University Hospital approved the research project. Parents of all participants signed informed consent. The data are anonymized.

The sample includes 37 individuals having undergone ALL treatment (males *n* = 19, females *n* = 18) aged 4 to 21 (the youngest 4.5 years old, the oldest 20.8 years old, 10.26 ± SD 3.9, median 9.5). They received their last vincristine (VCR) dose at least six months and no longer than six years ago. We contacted the patients at the Department of Pediatric Hematology and Oncology, 2nd Medical Faculty, Charles University, and Motol University Hospital in Prague. All participants were treated according to the AIEOP-BFM ALL 2009 treatment protocol. Patients with relapsed disease, patients after bone marrow transplantation, those referred for bone marrow transplantation, patients with peripheral neuropathy of aetiology other than vincristine-induced peripheral neuropathy (VIPN), and patients with mental disabilities (e.g,. Down syndrome) were not included in the study.

In the current study we used BOT-2 CF (31, 39). It consists of 53 motor performance items divided into eight subtests: 1) Fine Motor Precision, 2) Fine Motor Integration, 3) Manual Dexterity, 4) Upper-Limb Coordination, 5) Bilateral Coordination, 6) Balance, 7) Running Speed and Agility, and 8) Strength; and four composite areas: I. Fine Manual Control, II. Manual Coordination, III. Body Coordination, IV. Strength and Agility. BOT-2 SF contains 14 items selected from each sub-test from BOT-2 CF. To adequately compare the results of the SF and CF, we used the standard score for the CF and the standard score for the SF. Both scores represent normalized values, which consider the age and sex of the participants (31).

Professionally trained physiotherapists tested the patients at the children's section of the Department of Rehabilitation and Sports Medicine of the 2nd Medical Faculty of Charles University and Motol University Hospital in the morning hours from November 2019 to October 2021. Testing one person by BOT-2 CF took 50–70 min. The results were recorded in printed form. Subsequently, we overwrote the data in Pearson's online program, Q-global. Data for BOT-2 SF were extracted from BOT-2 CF results (31).

## 3. Data extraction, statistical analysis

The Anderson-Darling and Kolmogorov-Smirnov tests did not reject the normality in BOT-2 CF. In contrast, in BOT-2 SF, the Kolmogorov-Smirnov test rejected the normality due to the presence of one outlier, a highly above-average standard score. Box's M Test shows passing circularity and covariance matrices equality. Therefore, considering the robustness of the analysis of variance for such violation of normality (40, 41), we used repeated measures ANOVA to compare the standard scores of BOT-2 CF and BOT-2 SF depending on the participants' sex and to determine statistical significance at *p* < 0.05. In addition, the study sample is design selected. Therefore, we calculated effect size (ES) (Hays *ω*^2^) with the following cutoffs: Hays *ω*^2^ = 0.01**–**0.07 small effect; Hays *ω*^2^ = 0.071**–**0.14 medium effect; Hays *ω*^2^ > 0.14 large effect (42). To analyze the sensitivity and specificity of BOT-2 SF, we used the Receiver Operating Characteristic (ROC) analysis, including the Area Under the Curve (AUC) parameter (43). The clinical usefulness of BOT-2 SF was defined as sensitivity + specificity greater than 1.5 (44) along with AUC < 0.7 (45). We used the Intraclass Correlation Coefficient (ICC) to approximate the reliability and uniformity of results between the BOT-2 sub-test composite scores and standard scores from BOT-2 CF, respectively BOT-2 SF. We followed the guidelines to interpret ICC: < 0.5 poor reliability; 0.5–0.75 moderate reliability; 0.75–0.90 good reliability; > 0.90 excellent reliability (46). The size effect (ES) of differences between two ICCs was interpreted as the ES magnitude: small effect, Cohen *q* = 0.1–0.3; medium effect, Cohen q = 0.3–0.5; large effect, Cohen q > 0.5. The data were evaluated by the NCSS2007 program (Version 2007; NCSS, Kaysville, UT, United States).

## 4. Results

The results of repeated measures ANOVA controlling for the sex of participants show that participants achieved a significantly lower standard score in BOT-2 SF = 45.1 (± 7.9) in comparison to BOT-2 CF = 49.1 (± 9.4) F (1, 35) = 27.50, *p* < 0.001 with large effect size (ES) Hays *ω*^2^ = 0.41.

Males outperform females in all BOT-2 composites and BOT-2 CF and BOT-2 SF standard scores. However, the main effect of sex was not proved, F (1, 35) = 1.36, *p* = 0.25, Hays *ω*^2^ = 0.009 (Table 1). Nevertheless, in the Fine Motor Control composite score, we found a significant difference in variability of results (in the values of standard deviations) according to sex F_36_ = 2.40 *p* < 0.05. Girls performed in this composite subtest non-significantly worse but were significantly more homogeneous in their performance compared to boys.

**Table 1:** Results of BOT-2 CF and BOT-2 SF, boys, girls.

Composite	Boys *n* = 19	Girls *n* = 18	The difference in scores between boys and girls
	Mean ± SD	Mean ± SD	Mean dif–CI95%
Fine Manual Control	53.3 ± 12.05	51.8 ± 7.8	1.5 (−5.32 – + 8.30)
Manual Coordination	49.6 ± 9.1	45.8 ± 8.1	3.2 (−2.48 – + 9.00)
Body Coordination	55.3 ± 9.8	53.1 ± 9.8	2.2 (−4.31 – + 8.73)
Strength and Agility	46.2 ± 10.5	42.9 ± 9.3	3.3 (−3.35– + 9.89)
BOT-2 CF	50.6 ± 10.9	47.4 ± 7.6	3.2 (−3.11– + 9.49)
BOT-2 SF	46.7 ± 8.7***	43.4 ± 6.9***	3.3 (−2.02– + 8.50)

SD, standard deviation.

*** *p* < 0.001 in boys and girls the standard scores between BOT-2 CF and BOT-2 SF differ significantly.

Furthermore, we found the most significant difference in the composite Manual Coordination, where females scored about 3.8 standard points worse than males. In addition, both males and females perform the worst in the composite Strength and Agility, which assesses aspects of physical readiness–physical fitness (PF).

The ICC between BOT-2 CF and BOT-2 SF is 0.78. Furthermore, the uniformity of the results (the stability) between BOT-2 CF and BOT-2 SF shows “good” reliability when controlling for sex, in boys ICC = 0.78 and girls ICC = 0.76.

In the next step, we compared with the ICC the uniformity of results between composite standard scores and standard scores of BOT-2 CF and BOT-2 SF. We found the most significant differences in ICC values between standard scores BOT-CF and BOT-SF and the Body Coordination composite with more robust evidence in boys. In addition, regardless of the sex of participants, the Fine Manual Control composite and Body Coordination composite had the weakest uniformity with the BOT-2 CF and BOT-2 SF standard score. The main reason for this finding is the very low ICC between the Fine Manual Control composite and standard scores from both forms of BOT-2 girls. It would mean that the Fine Manual Control level and Body Coordination level in ALL girls has low reliability and is poorly related to overall motor proficiency. Separate ICC analyses showed a similar lack of uniformity between composites and BOT-2 CF and BOT-2 SF with large ES: Body Coordination, medium ES: Strength and Agility or small ES: Manual Coordination. The highest uniformity with “good” reliability was found between BOT-2 CF and BOT-2 SF standard scores and the composite of Strength and Agility (Table 2).

**Table 2:** Intraclass correlation coefficient differences between composite scores and BOT-2 CF and BOT-2 SF standard scores–total, boys, girls.

Composite	BOT-2 CF standard score (boys and girls)	BOT-2 SF standard score (boys and girls)	Cohen q
Fine Manual Control	0.60	0.26	0.44**
Manual Coordination	0.74	0.65	0.14*
Body Coordination	0.67	0.29	0.51***
Strength and Agility	0.73	0.86	0.22*
	BOT-2 CF standard score girls	BOT-2 SF standard score girls	
Fine Manual Control	0.25	0.02	0.24*
Manual Coordination	0.69	0.65	0.02
Body Coordination	0.60	0.25	0.44**
Strength And Agility	0.66	0.80	0.12*
	BOT-2 CF standard score boys	BOT-2 SF standard score boys	
Fine Manual Control	0.77	0.49	0.48*
Manual Coordination	0.77	0.64	0.25*
Body Coordination	0.72	0.32	0.58***
Strength and Agility	0.77	0.89	0.28*

Significance of ICC differences between CF and SF: *small ES, Cohen *q* = 0.1–0.3; ** medium ES, Cohen *q* = 0.3–0.5; *** large ES, Cohen *q* > 0.5.

Furthermore, we wanted to know if BOT-2 SF can categorize participants with their final score with sufficient sensitivity and specificity compared to the categorization obtained from BOT-2 CF. Therefore, we also analysed the sensitivity and specificity of BOT-2 SF. We used calculated Kendal's Tau correlation between the categorization of participants obtained from BOT-2 CF and BOT-2 SF, which is Kendal Tau = 0.81. We worked with a five-point Likert scale established according to Bruininks and Bruininks' manual (31) in the ROC analysis procedure. In both BOT-2 CF and BOT-2 SF, this scale transforms the standard score into final categories (1—well below-average; 2—below-average; 3—average; 4—above-average; 5—well above-average) (30). According to the ROC analysis assessing sensitivity and specificity, BOT-2 SF obtained acceptable sensitivity (72.3%) and high specificity (91.9%) with high accuracy of 86.1%, and the fair value of Empirical AUC = 0.734 CI95% (0.47-0.88) in comparison to BOT-2 CF. BOT-SF can replicate motor proficiency with as high probability as BOT-2 CF but systematically underestimate motor proficiency.

## 5. Discussion

This study fills the gap in observational methods and compares the compatibility of BOT-2 CF and BOT-2 SF in ALL survivors. Verifying the substitutability and usability of BOT-SF would simplify the diagnosis of essential areas of motor proficiency related to daily living activities (ADL) and strengthen the child's health and self-sufficiency (1). We use the standard scores considering age and sex for comparing results from the SF and CF (29).

Firstly, the standard score obtained from BOT-2 SF showed “good reliability” with the standard score from BOT-2 CF in both sexes, boys ICC = 0.78, *r* = 0.86 and girls ICC = 0.76. Furthermore, the expected score from both versions strongly correlated with *r* = 0.90. Therefore, BOT-2 SF seems to assess the same construct of motor proficiency with acceptable reliability when compared with BOT-2 CF. These results correspond with the previous findings of Bruininks and Bruininks (29), Mancini et al. (35) and Jirovec et al. (31).

Although BOT-2 SF has acceptable reliability and strongly correlates with BOT-2 CF, it significantly underestimates the level of motor proficiency (SF = 45.1 ± 7.9, CF = 49.1 ± 9.4) in children and young adults—both male and female—after ALL treatment. It aligns with the conclusion of Jirovec et al. (33) on healthy school children. Still, it contradicts Mancini et al. (35), who found in children with ADHD that BOT-2 SF overestimates the level of motor proficiency compared to BOT-2 CF. The explanation for the different findings could be that BOT-2 CF is much more time-consuming (45–60 min per participant) and requires close attention from the participant. Therefore, ADHD children with impaired attention might have difficulty concentrating throughout the entire BOT-2 CF testing procedure.

There are several reasons for the overestimation or underestimation of the assessed construct/unobserved latent variables. In test development, one assumption is that for adequate discrimination power and accuracy of the test battery, we must have an appropriate number of indicators with fair convergent and divergent item validity (47). Furthermore, these indicators' factorial validity for the assessed construct should be verified. Also the indicators' local independence and the lowest number of indicators still passing the acceptable value of approximated generic reliability should be known (48). BOT-2 CF has 53 items; previous studies verified that this battery has a stable four factors structure (29). Conversely, BOT-2 SF with 14 items is presented as a one-factor or unidimensional screening test battery. Recent studies on populations of children with neurotypical development supported the claim that BOT-2 SF assesses one theoretical construct, namely motor proficiency (34) or motor competency (49). However, Bardid et al. (48) added that many items from BOT-2 SF showed unordered threshold parameters, indicating that the categories in motor proficiency were not related to a child's performance level. Brown's study reached a similar conclusion (35). Authors noted that 9 out of 14 items in BOT-2 SF did not meet psychometric requirements such as item difficulty or acceptable factorial validity (35). In addition, Jirovec et al. (33) suggested that fewer indicators could result in lower standard scores obtained from BOT-2 SF because of the difficulty of selected items. In the BOT-2 manual (29), there is no information on how these 14 items for BOT-2 SF were selected. We have a statement that each sub-test assess maximum of two tests in BOT-2 SF. Furthermore, some sub-tests from BOT-2 SF estimate the proficiency only from one directly measured item (Manual Dexterity, Running Speed and Agility, Strength). However, using a single indicator to assess the unobserved latent variable contradicts test development rules, which recommend using at least three indicators per latent variable (49–51). Moreover, based on the generalizability theory (G-theory), the number of valid indicators used in a test or test battery is directly linked to the accuracy with which we estimate the assessed trait (52). Therefore, fewer indicators in BOT-2 SF might provide limited information about the level of motor proficiency.

As do Jirovec et al. (31), we provide support for this assumption in our study based on detailed correlation analyses between standard scores of BOT-2 SF, BOT-2 CF, and BOT-2 CF composite scores achieved in four motor domains: Fine Manual Control, Manual Coordination, Body Coordination, Strength and Agility. Composite scores from four motor domains explained *R*^2^ = 57% of motor proficiency estimated with BOT-2 CF as opposed to *R*^2^ = 44% of motor proficiency assessed with BOT-2 SF. The most significant differences in the reliability of the results (low stability and predictability) between the composite's standard scores of BOT-2 CF and BOT-2 SF are for the Fine Manual Control and Body Coordination composites, with more significant differences for boys. The BOT-2 SF standard scores had significantly weaker uniformity with Body Coordination composite score than BOT-2 CF. There is also a question of whether BOT-2 CF contains more items with low difficulty (ceiling effect items) or whether the content similarity of items does not allow any possible violation of the local independence, which means significant correlations between item errors.

Regardless of sex, the Fine Manual Control has the weakest ICC with BOT-2 CF (ICC = 0.60) and BOT-2 SF (ICC = 0.26). Weak reliability was markedly lower in girls (BOT-2 CF, ICC = 0.25, and BOT-2 SF, ICC = 0.02). It could indicate that the level of Fine Manual Control in girls has low reliability and is poorly related to overall motor proficiency. In contrast, Jirovec et al. (31) revealed the lowest ICC (*r* = 0.65) between the CF standard score and Strength and Agility. In our results, Strength and Agility show the highest reliability of the four composites with CF (ICC = 0.73) and SF (ICC = 0.86) across sex. The different findings between our study and Jirovec et al. may be due to a spectrum of participants. We measured patients after ALL with possibly decreased levels of PF after the treatment. Our sample achieved $\bar{x}$ = 44.5 of composite score in Strength and Agility while participants in the study by Jirovec et al., healthy children, achieved $\bar{x}$ = 49 composite scores in Strength and Agility. Our study found the most significant differences between ICCs for Body Coordination and Fine Manual Control and BOT-2 CF and BOT-2 SF standard scores. ICCs calculated from Body Coordination composite score, and BOT-2 SF standard score and Fine Manual Control composite score and BOT-2 SF standard score, are significantly lower than BOT-2 CF with medium and large ES. The differences in ICCs between BOT-2 CF and SF standard scores and the Body Coordination composite are slight for both sexes. Jirovec et al. (31) reported a similar finding only in girls. However, in Fine Motor Control, the differences in ICCs are sex-dependent. In girls, the results from Fine Motor Control hardly match the overall standards score achieved from BOT-2 SF. We assume that this poor uniformity has the cause of the low variability of results in girls. Since all correlation analysis is built upon variances, the significantly narrower standard deviation could decrease the uniformity calculated as ICC. Other results from our study generate differences among composites and CF and SF ICCs with small to medium ES.

In previous studies, Brahler et al. (26) and Carmosino et al. (27) attempted a more detailed analysis of the relationships between each item and subtests from BOT-2. Brahler et al. examined the Strength, Balance, Fine Motor Precision, and Fine Motor Integration subtests. Carmosino et al. analyzed Manual Dexterity, Upper-Limb Coordination, Bilateral Coordination, Running Speed, and Agility subtests. Both studies aimed to determine the association between the items in the four subtests and the total score. Brahler et al. (26) found a wide range of item correlations with the subtests Total Point Score (TPS) (*r* = 0.07 to 0.86). A follow-up study by Carmosino et al. (27) found that all test items of the three subtests (Manual Dexterity, Upper-Lib Coordination, Running Speed and Agility) correlate with the subtests TPS. They found that not all items in the SF have the highest correlation among the given subtest items. The high correlation between the items and the subtest's TPS in the CF and SF does not necessarily mean that the two versions provide comparable or identical motor proficiency results. Surprisingly these two studies pay little attention to the difference in the total score of BOT-2 CF and SF.

The studies mentioned above investigated the item's point score and the subtest's TPS. The question is whether these are meaningful comparisons and statistical calculations, as the authors did not take age and sex into account. At the same time, each item's point score has a different maximum point. SF provides only an overall result, and there is no possibility of analysing individual motor components within the normative data. The BOT-2 CF processing form and its manual do not allow us to divide it into individual subtests, so BOT-2 SF cannot be divided into unique TPS for individual subtests (probably due to the small number of items within the subtest). Therefore, we only compared the total standard score of BOT-2 CF with BOT-2 SF. However, we can use the individual items and subtests of the SF in clinical praxis, but only to evaluate the child's results over time.

Regarding the performance in the four motor composites, our patients were most significantly lacking in the Strength and Agility motor composite with 40.5% below-average and well below-average results (with 54.1% average and 5.4% above and well above-average results). In addition, patients performed the worst in the Running Speed and Agility subtest, with 59.5% below-average and well below-average results (with 37.8% average and 2.7% above and well above-average results). Patients also had motor difficulties in the Manual Coordination motor composite, with 21.6% below-average and well below-average results (with 70.3% average and 8.1% above average). Furthermore, they displayed the most significant shortcomings in the subtest Manual Dexterity with 16.2% below-average and well below-average results (with 78% average and 5.4% above average).

In contrast, 27% of patients achieved well above-average and above-average results in the Fine Manual Control motor composite (with 67,6% average and 5,4% below and well below-average results), with the best performance in the subtest Fine Motor Integration at 32,4% well above-average and above-average results (with 54,1% average and 13,5% below and well below-average results). Similarly, 24,3% of patients performed well above average and above average in the Body Coordination motor composite (with 64,9% average and 10,8% below and well below-average results). In this motor composite, patients performed the best in the Bilateral Coordination subtest with 27% well above-average and above-average results (with 64,9% average and 8,1% below and well below-average).

These results showed that ALL and its treatment negatively impact the Strength and Agility of the patients. A period of growth and development of basic motor skills, coordination, and strength is suddenly disrupted by a disease associated with frequent hospitalizations and demanding treatment, which can impact the cardiorespiratory system. It affects the amount of physical activity patients do and their relationship to it. This, in turn, affects their health and quality of life. On the other hand, the Fine Manual Control was mostly average to above average for the given population, which is consistent with the study by Nama et al. (53). This can be attributed to patients orientating mainly to manual activities during hospitalization in bed and during periods of significant fatigue. Different conclusions were reached by Hanna et al. (22) and Kabak et al. (2) regarding motor deficit in Fine Manual Control in patients with ALL. Compared with healthy individuals, Hanna et al. (22) found significantly worse performance in the Fine Manual Control assessed by BOT-2 CF in ALL patients than in healthy control in maintenance therapy. However, they investigated children between the ages of 4 and 7, when Fine Manual Control starts to develop, so the onset of ALL at this age can fundamentally affect the development of given motor skills. At the same time, during our protocol, we did not assess the level of neuropathy, which causes fine motor problems and which is usually more extensive in patients during maintenance therapy compared to survivors (49), and causes fine motor problems.

Using ROC analysis, we evaluated the sensitivity and specificity of BOT-2 SF BOT-2 SF showed acceptable sensitivity (72.3%) and high specificity (91.9%) with high accuracy (86.1%). Our results differ from those of Jirovec et al. (31) in that high sensitivity (84%) but low specificity (42.9%) were reported. Jirovec et al. concluded that BOT-2 SF is a valuable tool for detecting motor deficits but not for testing psychometrically above-average individuals. Our results suggest that BOT-2 SF can replicate the level of motor proficiency of BOT-2 CF with high probability. Mancini et al. (35) found an SF sensitivity of 69.23% for identifying children at the 17th percentile and below (i.e., the below-average and well below-average categories). The specificity, or the chance the SF will rate an individual as non-threatened when the CF evaluates individual as non-threatened, is 100% for the 17th percentile and below.

From the findings mentioned above, we could conclude that BOT-2 SF is helpful when we want to discover whether motor proficiency in ALL patients is below average or above-average. Nevertheless, we must consider that BOT-2 SF shows a worse standard score compared to BOT-2 CF. Therefore, BOT-2 SF is stricter in assessing motor proficiency performance. It means that BOT-2 SF might interpret the motor proficiency of ALL patients as below average even though BOT-2 CF would identify them as average. In the case of a below-average score from BOT-2 SF, we suggest testing ALL patients with BOT-2 CF (whole or parts) to find out in which areas of motor proficiency the patient has the most significant difficulties. Furthermore, it seems the PF level of ALL patients significantly influences the final standard score of BOT-2 SF. Therefore, we highly recommend including PF exercise for rehabilitation during ALL treatments.

The limitations of this study were the small sample size and the time since treatment ended. However, the reliability of the measurement was strong. To evaluate performance, it is necessary first to assess whether the individual corresponds to developmental calendar age (CA), as most standards are based on CA. On the other hand, biological age (BA) refers to the human functional capacity and can differ considerably between individuals of the same CA (54). If the BA is significantly different from the CA, it is critical to approach the participant individually and assess their performance according to modified standards. Differences compared to CA can be up to ± three years (54, 55). The treatment can also affect the BA. It would be interesting to know the motor maturity of the given individual before and after treatment and how it changes because of the treatment. However, this is a subject for further study.

## 6. Conclusions

BOT-2 SF seems to be a helpful tool for assessing motor proficiency in patients after ALL treatment. BOT-2 SF replicates motor proficiency with the same high degree of probability as BOT-2 CF but systematically underestimates the level of motor proficiency. The question remains whether the items in BOT-2 SF chosen from the BOT-2 CF items have the highest validity for assessing motor proficiency in the population after ALL treatment. ALL patients performed the worst in Strength and Agility. Therefore, we suggest systematically applying suitable PF exercises in ALL patients. We recommend testing with BOT-2 CF to find out the most significant motor difficulties.

## Data Availability

The original contributions presented in the study are included in the article Further inquiries can be directed to the corresponding author.

## References

[1] Hunger SP Lu X Devidas M Camitta BM Gaynon PS Winick NJ Improved survival for children and adolescents with acute lymphoblastic leukemia between 1990 and 2005: a report from the Children's Oncology group. J Clin Oncol. (2012) 30(14):1663–9. 10.1200/JCO.2011.37.8018 22412151 PMC3383113

[2] Yildiz Kabak V Ekinci Y Atasavun Uysal S Cetin M Duger T. Motor and basic cognitive functions in children with acute lymphoblastic leukemia undergoing induction or consolidation chemotherapy. Percept Mot Skills. (2021) 128(3):1091–106. 10.1177/00315125211002065 33730934

[3] Folber F Doubek M. Advances in treatment of acute lymphoblastic leukemia in adults. Onkologie. (2013) 7(3):113–6.

[4] Margolin JF. Acute lymphoblastic leukemia. Principle and Practice of Pediatric Oncology. (1997):316–367.

[5] Starý J. Akutní leukemie u dětí. Onkologie. (2010) 4(2):120–4.

[6] Kaste SC Rai SN Fleming K McCammon EA Tylavsky FA Danish RK Changes in bone mineral density in survivors of childhood acute lymphoblastic leukemia. Pediatr Blood Cancer. (2006) 46(1):77–87. 10.1002/pbc.20553 16106430

[7] Mäkitie O Heikkinen R Toiviainen-Salo S Henriksson M Puukko-Viertomies LR Jahnukainen K. Long-term skeletal consequences of childhood acute lymphoblastic leukemia in adult males: a cohort study. Eur J Endocrinol. (2013) 168(2):281–8. 10.1530/EJE-12-0702 23197573

[8] Onciu M. Acute lymphoblastic leukemia. Hematol Oncol Clin North Am. (2009) 23(4):655–74. 10.1016/j.hoc.2009.04.009 19577163

[9] De Luca CR McCarthy M Galvin J Green JL Murphy A Knight S Gross and fine motor skills in children treated for acute lymphoblastic leukaemia. Dev Neurorehabil. (2013) 16(3):180–7. 10.3109/17518423.2013.771221 23477341

[10] Ratei R Basso G Dworzak M Gaipa G Veltroni M Rhein P Monitoring treatment response of childhood precursor B-cell acute lymphoblastic leukemia in the AIEOP-BFM-ALL 2000 protocol with multiparameter flow cytometry: predictive impact of early blast reduction on the remission status after induction. Leukemia. (2009) 23(3):528–34. 10.1038/leu.2008.324 19020543

[11] Schrappe M. International Collaborative Treatment Protocol For Children And Adolescents With Acute Lymphoblastic Leukemia. clinicaltrials.gov; 2020 October (citated 19. April 2022). Report No.: NCT01117441. Available at: https://clinicaltrials.gov/ct2/show/NCT01117441

[12] Joyce ED Nolan VG Ness KK Ferry RJ Jr Robison LL Pui CH Association of muscle strength and bone mineral density in adult survivors of childhood acute lymphoblastic leukemia. Arch Phys Med Rehabil. (2011) 92(6):873–9. 10.1016/j.apmr.2010.12.039 21621662 PMC3321361

[13] San Juan AF Fleck SJ Chamorro-Vina C Maté-Muñoz JL Moral S Garcia-Castro J Early-phase adaptations to intrahospital training in strength and functional mobility of children with leukemia. J Strength & Cond Res. (2007) 21(1):173–7. 10.1519/00124278-200702000-00031 17313277

[14] Perondi MB Gualano B Artioli GG de Salles Painelli V Filho VO Netto G Effects of a combined aerobic and strength training program in youth patients with acute lymphoblastic leukemia. J Sports Sci Med. (2012) 11(3):387–92. 24149344 PMC3737942

[15] White J Flohr JA Winter SS Vener J Feinauer LR Ransdell LB. Potential benefits of physical activity for children with acute lymphoblastic leukaemia. Pediatr Rehabil. (2005) 8(1):53–8. 10.1080/13638490410001727428 15799136

[16] Le Meignen M Auquier P Barlogis V Sirvent N Contet A Simeoni MC Bone mineral density in adult survivors of childhood acute leukemia: impact of hematopoietic stem cell transplantation and other treatment modalities. Blood, the J Am Soci Hematol. (2011) 118(6):1481–9. 10.1182/blood-2011-01-332866 21596857

[17] Maniadaki I Stiakaki E Germanakis I Kalmanti M. Evaluation of bone mineral density at different phases of therapy of childhood all. Pediatr Hematol Oncol. (2006) 23(1):11–8. 10.1080/08880010500313272 16326407

[18] Ness KK Kaste SC Zhu L Pui CH Jeha S Nathan PC Skeletal, neuromuscular and fitness impairments among children with newly diagnosed acute lymphoblastic leukemia. Leuk Lymphoma. (2015) 56(4):1004–11. 10.3109/10428194.2014.944519 25030039 PMC4336225

[19] Janiszewski PM Oeffinger KC Church TS Dunn AL Eshelman DA Victor RG Abdominal obesity, liver fat, and muscle composition in survivors of childhood acute lymphoblastic leukemia. J Clin Endocrinol Metab. (2007) 92(10):3816–21. 10.1210/jc.2006-2178 17652222

[20] Ness KK Baker KS Dengel DR Youngren N Sibley S Mertens AC Body composition, muscle strength deficits and mobility limitations in adult survivors of childhood acute lymphoblastic leukemia. Pediatr Blood Cancer. (2007) 49(7):975–81. 10.1002/pbc.21091 17091482

[21] Wuang YP Lin YH Su CY. Rasch analysis of the bruininks–oseretsky test of motor proficiency-in intellectual disabilities. Res Dev Disabil. (2009) 30(6):1132–44. 10.1016/j.ridd.2009.03.003 19395233

[22] Hanna S Elshennawy S El-Ayadi M Abdelazeim F. Investigating fine motor deficits during maintenance therapy in children with acute lymphoblastic leukemia. Pediatr Blood Cancer. (2020) 67(7):e28385. 10.1002/pbc.28385 32400963

[23] Ramchandren S Leonard M Mody RJ Donohue JE Moyer J Hutchinson R Peripheral neuropathy in survivors of childhood acute lymphoblastic leukemia. J Peripher Nerv Syst. (2009) 14(3):184–9. 10.1111/j.1529-8027.2009.00230.x 19909482 PMC3865985

[24] Montgomery PC Connolly BH. Norm-referenced and criterion-referenced tests: use in pediatrics and application to task analysis of motor skill. Phys Ther. (1987) 67(12):1873–6. 10.1093/ptj/67.12.1873 3685115

[25] Wang HY Long IM Liu MF. Relationships between task-oriented postural control and motor ability in children and adolescents with down syndrome. Res Dev Disabil. (2012) 33(6):1792–8. 10.1016/j.ridd.2012.05.002 22699252

[26] Brahler CJ Donahoe-Fillmore B Mrowzinski S Aebker S Kreill M. Numerous test items in the complete and short forms of the BOT-2 do not contribute substantially to motor performance assessments in typically developing children six to ten years old. J Occup Ther Sch & Early Interv. (2012) 5(1):73–84. 10.1080/19411243.2012.674746

[27] Carmosino K Grzeszczak A McMurray K Olivo A Slutz B Zoll B Test items in the complete and short forms of the BOT-2 that contribute substantially to motor performance assessments in typically developing children 6-10 years of age. J Stud Phys Ther Res. (2014) 7(2):31–43.

[28] Radanović D Đorđević D Stanković M Pekas D Bogataj Š Trajkovic N. Test of motor proficiency second edition (BOT-2) short form: a systematic review of studies conducted in healthy children. Children. (2021) 8(9):787. 10.3390/children8090787 34572219 PMC8471722

[29] Bruininks RH Bruininks BD. Bruininks-Oseretsky test of motor proficiency. Minnesota: AGS Publishing (2005).

[30] Bruininks RH Bruininks BD. Bruininks–oseretsky test of motor proficiency–manual. Circle pines, Minnesota, USA: American Guidance Service (1978).

[31] Jírovec J Musálek M Mess F. Test of motor proficiency second edition (BOT-2): compatibility of the complete and short form and its usefulness for middle-age school children. Front Pediatr. (2019) 7:153. 10.3389/fped.2019.00153 31065548 PMC6489893

[32] Wuang YP Su CY. Reliability and responsiveness of the bruininks–oseretsky test of motor proficiency-in children with intellectual disability. Res Dev Disabil. (2009) 30(5):847–55. 10.1016/j.ridd.2008.12.002 19181480

[33] Jirovec J Holický J. Comparison of complete and short forms of the bruininks-Oseretsky test of motor proficiency, second edition (BOT-2) in middle age school children. Česká Kinantropologie. (2017) 21(1–2):60–8.

[34] Brown T. Structural validity of the bruininks-oseretsky test of motor proficiency–second edition brief form (BOT-2-BF). Res Dev Disabil. (2019) 85:92–103. 10.1016/j.ridd.2018.11.010 30502549

[35] Mancini V Rudaizky D Howlett S Elizabeth-Price J Chen W. Movement difficulties in children with ADHD: comparing the long-and short-form Bruininks–oseretsky test of motor proficiency—second edition (BOT-2). Aust Occup Ther J. (2020) 67(2):153–61. 10.1111/1440-1630.12641 31944320

[36] Cairney J Hay J Veldhuizen S Missiuna C Faught BE. Comparing probable case identification of developmental coordination disorder using the short form of the bruininks-Oseretsky Test of motor proficiency and the movement ABC. Child Care Health Dev. (2009) 35(3):402–8. 10.1111/j.1365-2214.2009.00957.x 19397603

[37] Nocera VG Wood AP Wozencroft AJ Coe DP. The test–retest reliability of the bruininks–oseretsky test of motor proficiency-short form in youth with down syndrome—a pilot study. Int J Environ Res Public Health. (2021) 18(10):5367. 10.3390/ijerph18105367 34069921 PMC8157598

[38] Yeh KK Liu WY Yang ML Liu CH Lien HY Chung CY. Sufficiency of the BOT-2 short form to screen motor competency in preschool children with strabismus. PloS one. (2021) 16(12):e0261549. 34928993 10.1371/journal.pone.0261549 PMC8687543

[39] Deitz JC Kartin D Kopp K. Review of the bruininks-oseretsky test of motor proficiency, (BOT-2). Phys Occup Ther Pediatr. (2007) 27(4):87–102. 10.1080/J006v27n04_06 18032151

[40] Ito PK. 7 Robustness of ANOVA and MANOVA test procedures. Handb Stat. (1980) 1:199–236. 10.1016/S0169-7161(80)01009-7

[41] Harwell MR Rubinstein EN Hayes WS Olds CC. Summarizing monte carlo results in methodological research: the one-and two-factor fixed effects ANOVA cases. J Educ Stat. (1992) 17(4):315–39. 10.3102/10769986017004315

[42] Grissom RJ Kim JJ. Effect sizes for research: Univariate and multivariate applications. New York: Routledge (2012).

[43] Altman DG Bland JM. Diagnostic tests 3: receiver operating characteristic plots. BMJ: Br Med J. (1994) 309(6948):188. 10.1136/bmj.309.6948.188 8044101 PMC2540706

[44] Power M Fell G Wright M. Principles for high-quality, high-value testing. BMJ: Br Med J Evi-Based Med. (2013) 18(1):5–10. 10.1136/eb-2012-100645 PMC3585491 22740357

[45] Mandrekar JN. Receiver operating characteristic curve in diagnostic test assessment. J Thorac Oncol. (2010) 5(9):1315–6. 10.1097/JTO.0b013e3181ec173d 20736804

[46] Koo TK Li MY. A guideline of selecting and reporting intraclass correlation coefficients for reliability research. J Chiropr Med. (2016) 15(2):155–63. 10.1016/j.jcm.2016.02.012 27330520 PMC4913118

[47] Lane S Raymond MR Haladyna TM. Handbook of test development. Vol. 2. NY: Routledge New York (2016).

[48] McDonald RP. Test theory: A unified treatment. New Jersey: Psychology Press (2013).

[49] Bardid F Utesch T Lenoir M. Investigating the construct of motor competence in middle childhood using the BOT-2 Short form: an item response theory perspective. Scand J Med Sci Sports. (2019) 29(12):1980–7. 10.1111/sms.13527 31357223

[50] Reinders-Messelink HA van Weerden TW Fock JM Gidding CE Vingerhoets HM Schoemaker MM Mild axonal neuropathy of children during treatment for acute lymphoblastic leukaemia. Eur J Paediatr Neurol. (2000) 4(5):225–33. 10.1053/ejpn.1999.0310 11030069

[51] Freude G Jakob O Martus P Rose U Seibt R. Predictors of the discrepancy between calendar and biological age. Occup Med (Chic Ill). (2010) 60(1):21–8. 10.1093/occmed/kqp113 19666964

[52] Hagtvet KA Benson J. The motive to avoid failure and test anxiety responses: empirical support for integration of two research traditions. Anxiety Stress Coping. (1997) 10(1):35–57. 10.1080/10615809708249294

[53] Nama N Barker MK Kwan C Sabarre C Solimano V Rankin A Vincristine-induced peripheral neurotoxicity: a prospective cohort. Pediatr Hematol Oncol. (2020) 37(1):15–28. 10.1080/08880018.2019.1677832 31682156

[54] Malina RM Bouchard C Bar-Or O. Growth, maturation, and physical activity. Champaign: Human kinetics (2004).

[55] Bogin B. Patterns of human growth (vol. 88). Cambridge: Cambridge University Press (2020).

